# Epigenetic silencing of genes and microRNAs within the imprinted Dlk1-Dio3 region at human chromosome 14.32 in giant cell tumor of bone

**DOI:** 10.1186/1471-2407-14-495

**Published:** 2014-07-09

**Authors:** Burkhard Lehner, Pierre Kunz, Heiner Saehr, Joerg Fellenberg

**Affiliations:** 1 Research Centre for Experimental Orthopedics, Department of Orthopedics, Trauma Surgery and Paraplegia, Orthopedic University Hospital Heidelberg, Schlierbacher Landstr 200a, Heidelberg 69118, Germany

**Keywords:** Giant cell tumor, Mesenchymal stem cell, MicroRNA, Epigenetics, Methylation

## Abstract

**Background:**

Growing evidence exists that the neoplastic stromal cell population (GCTSC) within giant cell tumors (GCT) originates from mesenchymal stem cells (MSC). In a previous study we identified a microRNA signature that differentiates between these cell types. Five differentially expressed microRNAs are located within the Dlk1-Dio3 region on chromosome 14. Aberrant regulation within this region is known to influence cell growth, differentiation and the development of cancer. The aim of this study was to elucidate the involvement of deregulations within the Dlk1-Dio3 region in GCT pathogenesis.

**Methods:**

Quantitative gene and microRNA expression analyses were performed on GCTSCs and MSCs with or without treatment with epigenetic modifiers. Methylation analysis of differentially methylated regions was performed by bisulfite sequencing.

**Results:**

In addition to microRNA silencing we detected a significant downregulation of *Dlk1*, *Meg3* and *Meg8* in GCTSCs compared to MSCs. DNA methylation analyses of the Meg3-DMR and IG-DMR revealed a frequent hypermethylation within the IG-DMR in GCTs. Epigenetic modification could restore expression of some but not all analyzed genes and microRNAs suggesting further regulatory mechanisms.

**Conclusion:**

Epigenetic silencing of genes and microRNAs within the Dlk1-Dio3 region is a common event in GCTSCs, in part mediated by hypermethylation within the IG-DMR. The identified genes, micro RNAs and microRNA target genes might be valuable targets for the development of improved strategies for GCT diagnosis and therapy.

## Background

Although generally benign, giant cell tumors of bone (GCT) are characterized by a locally aggressive behavior. They represent about 5% of all primary bone tumors and are frequently located at the meta-epiphyseal region of long bones including the distal femur, proximal tibia and the radius [1,2]. GCTs induce expansive osteolytic defects associated with significant bone destruction. Despite their benign nature, GCTs are characterized by a highly variable and unpredictable behavior. Although rare, GCT can manifests a malignant phenotype, and metastases have been described in up to 5% of the cases [3,4]. The current treatment is restricted to surgical resection of the tumor, which is, however, associated with a high recurrence rate [5]. Histologically, GCTs consists of multinucleated giant cells, histiocytes and fibroblast-like stromal cells, which are supposed to represent the neoplastic cell population. A subpopulation of these neoplastic stromal cells (GCTSCs) are characterized by the expression of mesenchymal stem cell (MSC) markers including CD73, CD105 and CD166 as well as the mesenchymal marker FGFR3 (fibroblast growth factor receptor3) [6,7]. Together with the fact that these cells display a differentiation potential comparable to MSCs, these data strongly indicate that GCTSCs develop from MSCs. In agreement with this hypothesis, we observed highly similar gene and microRNA expression profiles of GCTSCs and MSCs in previous studies [8,9]. However, we could also identify a differentially expressed microRNA signature that separates GCTSCs from MSCs, suggesting possible roles of the identified microRNAs and their target genes in the development and progression of GCTs [9]. Interestingly, five of the identified, differentially expressed microRNAs are arranged within two microRNA clusters located on human chromosome 14q32 [10]. These microRNA clusters have already been shown to be downregulated in ovarian cancer, melanoma and gastrointestinal stromal tumors, suggesting an important role of the encoded microRNAs for the development of several types of tumors [11-13]. The microRNA clusters are located within an imprinted chromosomal region designated Dlk1-Dio3 locus that harbors several protein-coding, paternally expressed genes including *Dlk1* (delta-like homolog 1), *Rtl1* (retrotransposon-like 1) and *Dio3* (iodothyronine deiodinase 3) and the non-coding, maternally expressed genes *Meg3* and *Meg8*. Imprinting of the Dlk1-Dio3 locus is regulated by two differentially methylated regions (DMRs) termed IG-DMR and Meg3-DMR [14,15]. The results of our previous studies suggest that deregulations within the Dlk1-Dio3 locus might be implicated in GCT pathogenesis. Therefore, the aim of this study was to investigate the expression of genes and microRNAs located within the Dlk1-Dio3 region in MSCs and GCTSCs with or without treatment with epigenetic modifiers. Analysis of methylation frequencies within the IG-DMR and Meg3-DMR in GCTSCs compared to MSCs were performed to detect possible implications of epigenetic alterations on the expression of differentially expressed genes and microRNAs that might contribute to GCT pathogenesis.

## Methods

The studies were approved by the Ethics Committee of the University of Heidelberg and informed consent to analyze tumor tissue and to publish clinical details was obtained from all individuals included in the study. Patient characteristics are summarized in Table 1.

**Table 1:** Characteristics of GCT patients

**Patient ID**	**Age**	**Gender**	**Tumor localization**
GCT-1	21	f	Right femur
GCT-2	31	f	Left tibia
GCT-3	60	m	Right patella
GCT-4	33	f	Left femur
GCT-5	37	f	Right femur
GCT-6	63	m	Left humerus
GCT-7	33	m	Left radius
GCT-8	28	m	Right femur
GCT-9	29	m	Right femur
GCT-10	48	m	Right tibia

### Sample preparation and cell culture

Primary GCTSCs were isolated from tissue samples derived from tumor resections in our clinic. The tissue was mechanically cut in small pieces and digested with 1.5 mg/ml collagenase B (Roche Diagnostics, Mannheim, Germany) for 3 h at 37°C in Dulbecco’s Modified Eagle Medium (DMEM) (Lonza GmbH, Köln, Germany) containing 4.5 g/l glucose and supplemented with 10% fetal calf serum (FCS) (Biochrom, Berlin, Germany), and 100 U/ml penicillin/streptomycin (Lonza GmbH, Köln, Germany). Cells were collected by centrifugation, washed twice in PBS and cultured in DMEM. Twenty-four hours after plating, cells were carefully treated with Trypsin/EDTA (Lonza GmbH, Köln, Germany) leaving the giant cells attached in the culture flask. Detached cells were cultured for further 3 passages eliminating any remaining giant cells and histiocytes. MSCs were isolated from fresh bone marrow samples derived from the iliac crest. Cells were fractionated on a Ficoll-Paque Plus density gradient (Amersham Pharmacia, Uppsala, Sweden), and the low-density MSC-enriched fraction was washed and seeded in culture flasks. MSC culture medium consisted of DMEM high glucose (Lonza GmbH, Köln, Germany) 12.5% FCS, 1× NEAA (non-essential amino acids) (Life Technologies, Darmstadt, Germany), 50 μM 2-mercaptoethanol (Life Technologies, Darmstadt, Germany) and 4 ng/ml bFGF (basic fibroblast growth factor) (Merck Chemicals GmbH, Schwalbach, Germany). After 24–48 h, cultures were washed with PBS to remove non-adherent material. During expansion, medium was replaced twice a week. For the treatment of cells with epigenetic modifiers, cells were seeded at 25% confluence and cultured for 10 days in medium containing 10 μM 5-Aza-2′-deoxycytidine (Sigma, Deisenhofen, Germany), 3 mM phenylbutyric acid (Sigma, Deisenhofen, Germany) or both. Medium was replaced every 2 days. Controls were cultured in medium without supplements.

### RNA extraction

Total RNA was extracted using mirVana miRNA Isolation Kit (Invitrogen, Darmstadt, Germany). RNA concentrations and purity were determined with a NanoDrop ND-1000 spectrophotometer (Peqlab, Erlangen, Germany). Extracted RNA was used for both, miRNA expression and RT-qPCR gene expression analyses.

### RT-qPCR

First strand complementary DNA (cDNA) was synthesized from 1 μg of total RNA using 1 μl Omniscript (Qiagen, Hilden, Germany), 10 μM oligo-dT primer, 5 mM dNTPs and 10U RNaseOut (Invitrogen, Karlsruhe, Germany) for 2 h at 37°C in a total volume of 20 μl. RT-qPCR was performed in the real-time thermal cycler Mx3005p (Agilent Technologies, Waldbronn, Germany) in a total volume of 20 μl using Absolute QPCR SYBR Green mix (Thermo scientific, Dreieich, Germany) and 1 μl of cDNA as template. Samples were heated to 95°C for 15 minutes followed by 40 cycles of denaturation at 95°C for 15 seconds, annealing at 58°C for 20 seconds and extension at 72°C for 30 seconds. After the last cycle, a melting curve analysis was performed to verify the specificity of the amplified PCR products. Calculated gene expression was normalized on the basis of the expression of *RPL19* (ribosomal protein L19) in the corresponding sample. The following primers were used: *Dlk1*-F: 5′-GACGGGGAGCTCTGTGATAG-3′, *Dlk1*-R: 5′-TCATAGAGGCCATCGTCCA-3′, *Meg3*-F: 5′-ACGGGCTCTCCTTGCATC-3′, *Meg3*-R: 5′-GCTTCCATCCGCAGTTCTTC-3′, *Meg8*-F: 5′-TGTCGGAGGATCGTGTCAT-3′, *Meg8*-R: 5′-AATCTTCTAGAGCCCCAGATCC-3′, *Rtl1*-F: 5′-CTCCAGAGAGGTGGATGGTC-3′, *Rtl1*-R: 5′-GATTGATGTCCGGATGGACT-3′, *Dio3*-F: 5′-CGCACAGCCCCTAGAATAGT-3′, *Dio3*-R: 5′-GCCACTACTATTTCCCTACAGAGC-3′, CD163-F 5′-GAAGATGCTGGCGTGACAT-3′; CD163-R 5′-GCTGCCTCCACCTCTAAGTC-3′; CD34-F 5′-TGGCTATTTCCTGATGAATCG-3′; CD34-R 5′-TCCACCGTTTTCCGTGTAAT-3′; CSF1R-F 5′-TCTGGTCCTATGGCATCCTC-3′; *RPL19*-F: 5′-GTGGCAAGAAGAAGGTCTGG-3′, *RPL19*-R: 5′-GCCCATCTTTGATGAGCTTC-3′.

### RT-qPCR of microRNAs

Quantification of microRNA expression was done using the TaqMan MicroRNA Reverse Transkription kit from Applied Biosystems (Darmstadt, Germany) according to the manufacturer′s instructions. In brief, 10 ng of total RNA isolated with the mirVana miRNA Isolation Kit was subjected to cDNA sysnthesis using microRNA specific stem-loop primer. For RT-qPCR 1.5 μl of cDNA was used in a total volume of 20 μl containing microRNA specific primer and TaqMan probes. Samples were heated to 95°C for 10 min followed by 40 cycles of denaturation at 95°C for 15 sec and a combined annealing/extension step at 60°C for 60 sec. The reaction was carried out in the real-time thermal cycler M×3005p from Agilent Technologies. Calculated microRNA expression levels were normalized on the basis of the RNU6B expression in the corresponding sample. RNU6B is a small nuclear RNA frequently used as reference RNA for microRNA quantification.

### RT-PCR of Meg3 splice variants

First strand complementary DNA (cDNA) was synthesized from 1 μg of total RNA as described for RT-qPCR. Amplification of *Meg3* isoforms was performed using 2 μl cDNA as template, 0.25 μl PlatinumTaq polymerase (Invitrogen), 0.6 μl MgCl_2_ (50 mM), 0.4 μl dNTPs (10 mM each) and 0.5 μl of each primer (10 μM) in a total volume of 20 μl. The following primers were used: MEG3EX3-F 5′-ACGGGCTCTCCTTGCATC-3′, MEG3EX4-F 5′-CTGCTTCCTGACTCGCTCTA-3′, MEG3EX5F 5′-GGCTGCAGACGTTAATGAGG-3′, MEG3EX6F 5′-TGTCTCCATCTCCTGCCAAG-3′, MEG3EX8-R 5′-GCTTCCATCCGCAGTTCTTC-3′. Samples were incubated at 94°C for 3 min followed by 36 cycles of denaturation at 94°C for 15 s, annealing at 58°C for 30s and extension at 72°C for 45 s and a final extension step at 72°C for 7 min. PCR products were separated on a 1.6% agarose gel and visualized by ethidiumbromide staining.

### Copy number assay

Total cellular DNA was extracted using DNeasy Tissue kit (Qiagen) according to manufacturer’s protocol. DNA copy number of the cytoband 14q32.2b on chromosome 14 was quantified using TaqMan copy number assay (Life Technologies) and the primers Hs03874180_cn. The copy number of the genomic RNAse P region was used as reference.

### Bisulfite sequencing

For methylation analysis of the IG-DMR and the Meg3-DMR, total cellular DNA was extracted using DNeasy Tissue kit (Qiagen) according to manufacturer’s protocol. One μg of DNA was bisulfite treated using EpiTect Plus Bisulfite kit (Qiagen). DNA fragments covering the IG-DMR and the Meg3-DMR, respectively, were amplified by PCR using the following primers: IG-DMR-F: 5′-TGGGATTATAGGTATTATGTTTGGA-3′, IG-DMR-R: 5′-CACTACTAAAAACTACATTTAAACAA-3′, Meg3DMR-F 5′- GTTAGGGATTAATTTTTATGTGTTAG-3′, Meg3DMR-R 5′-CAAATTCTATAACAAATTACTCTAAC-3′.

The IG-DMR fragment (909 bp) harbors 31 CpG dinucleotides and the Meg3-DMR (819 bp) harbors 44 CpG dinucleotides. According to the sequence NT_026437.12 at NCBI Database the position of the analyzed IG-DMR sequence is 82.276.640 – 82.277.549 and that of the analyzed Meg3-DMR fragment is 82.291.515 – 82.292.333. PCR products were cloned into pCR4-TOPO vector using TOPO TA cloning kit (Life Technologies) and sequenced. Methylation was analyzed using BiQ-Analyzer software [16].

## Results

Isolation of GCTSCs used in this study was performed as described previously. In brief, tumor tissue was enzymatically digested and the cells were taken into culture. Stromal cells and histiocytes were removed by trypsinization, leaving the giant cells attached in the culture flask. Detached cells were further cultured for 3 passages until only the neoplastic stromal cells survived. To verify the purity of the isolated GCTSCs they were tested for the absence of the monocytic/histiocytic markers CD163 and CD34 and the absence of colony stimulating factor 1 receptor (CSF1R) expressed by giant cells by RT-qPCR as described earlier [8].

**Figure 1:**
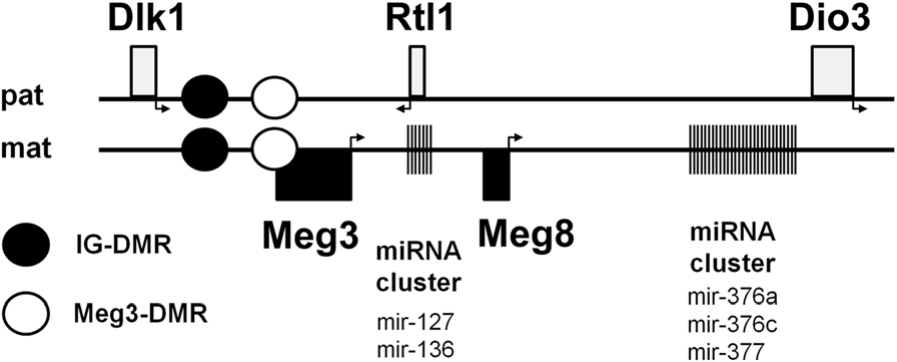
**Schematic illustration of the Dlk1-Dio3 locus on human chromosome 14q32.** The location of the noncoding maternally expressed genes *Meg3* and *Meg8*, the paternally expressed genes *Dlk1*, *Rtl1* and *Dio3*, the differentially methylated regions (DMRs) and the position of the microRNA clusters are indicated.

**Figure 2:**
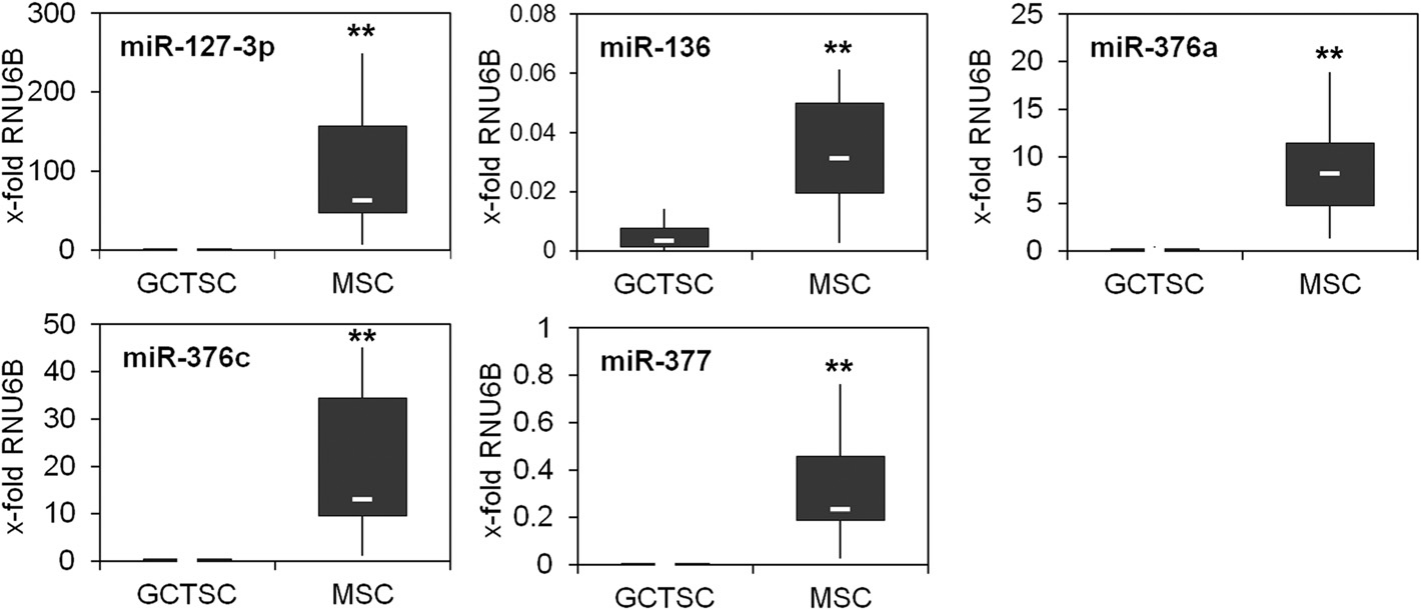
**Silencing of specific microRNAs in GCTSCs.** Total RNA including microRNAs was extracted from cultured GCTSCs (n = 10) and MSCs (n = 10) and expression of microRNAs was quantified relative to the expression of the small nuclear RNA RNU6B. The white lines indicate the median, the lower and upper boundaries of the box indicate the 25th and 75th percentile. The whiskers indicate the highest and lowest values. (**p < 0.01 determined by Mann–Whitney-*U* test).

**Figure 3:**
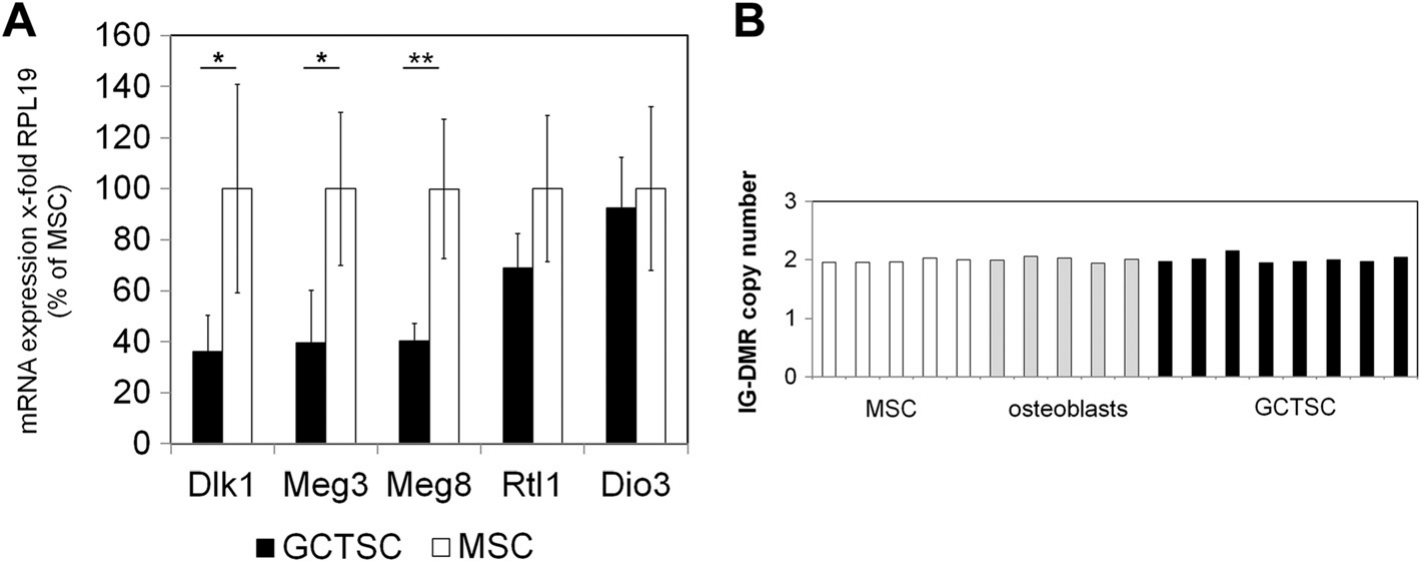
**Significant downregulation of *Dlk1*, *Meg3 *and *Meg8 *in GCTSCs. (A)** Expression of *Dlk1*, *Meg3*, *Meg8*, *Rtl1* and *Dio3* was analyzed by RT-qPCR in GCTSCs (n = 5) and MSCs (n = 5). Data were normalized on the basis of the ribosomal protein L19 (*RPL19*) expression in the corresponding sample. Data are presented as mean ± SD. (*p < 0.05 **p < 0.01 determined by Mann–Whitney-*U* test). **(B)** IG-DMR copy number assay. The IG-DMR copy number was determined by RT-qPCR in MSCs, GCTSCs and osteoblasts and calculated using the genomic RNAse P region as reference.

**Figure 4:**
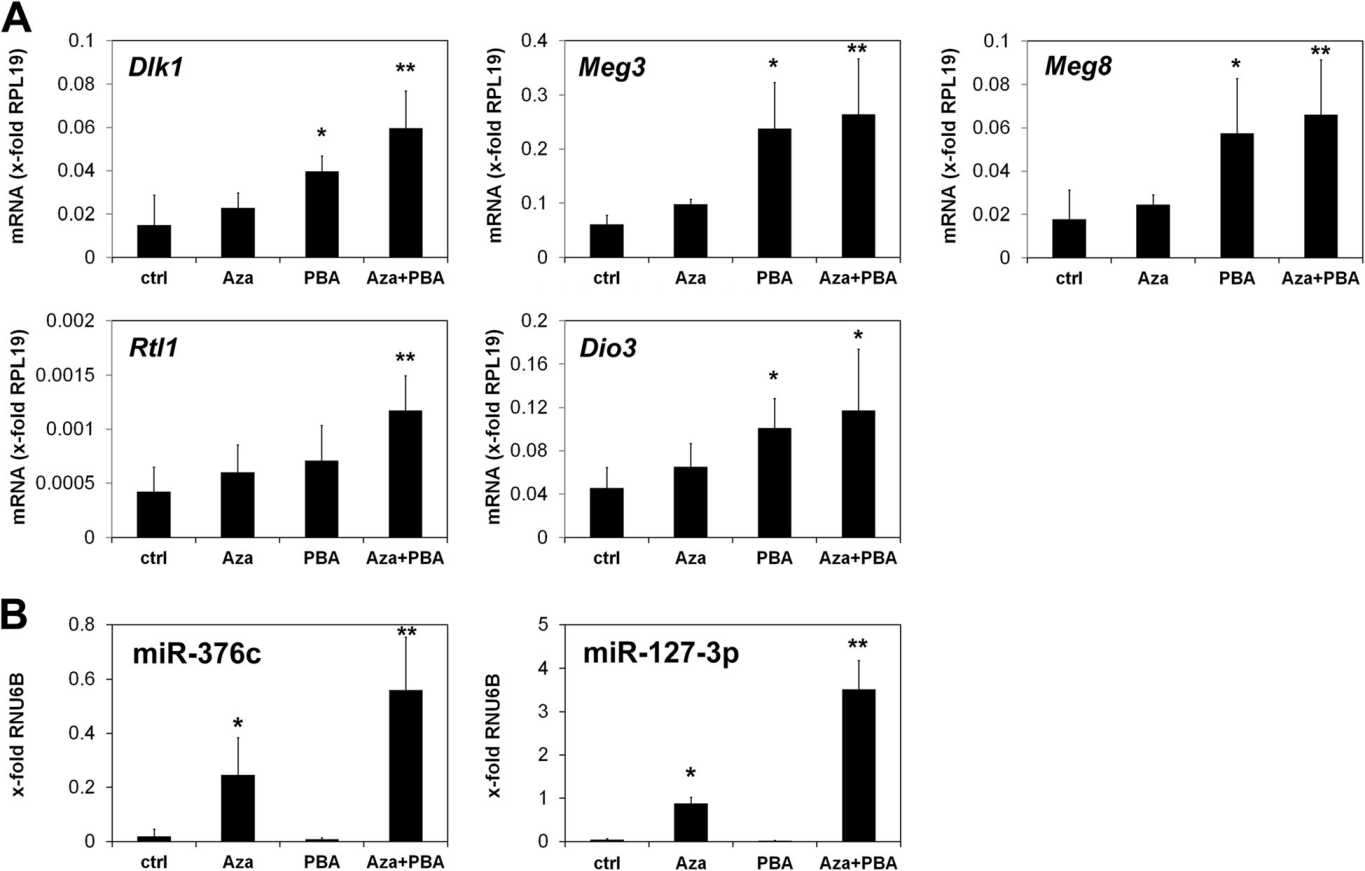
**Restoration of gene and microRNA expression in GCTSCs after treatment with epigenetic modifiers.** GCTSCs (n = 5) and MSCs (n = 5) were cultured in medium containing 10 μM 5-Aza-2′-deoxycytidine (Aza), 3 mM phenylbutyric acid (PBA) or both for 10 days. **(A)** Expression of *Dlk1*, *Meg3*, *Meg8, Rtl1* and *Dio3* normalized to the RPL19 expression in the corresponding sample. **(B)** Expression of miR-127-3p andmiR-376c normalized to the RNU6B expression in the corresponding sample. Data are presented as mean ± SD. (*p < 0.05 **p < 0.01 compared to untreated control cells determined by Mann–Whitney-*U* test).

**Figure 5:**
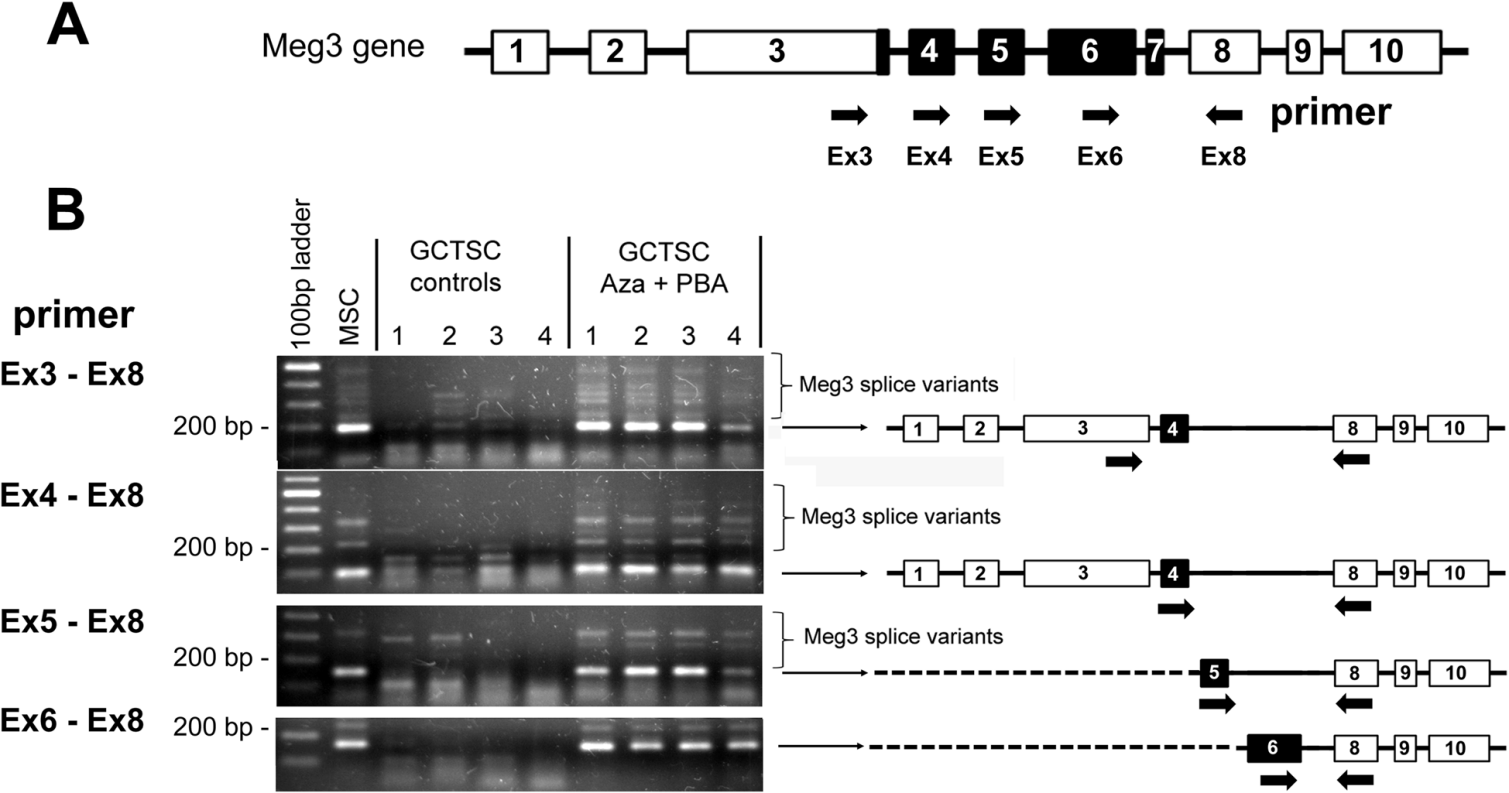
**Identification of Meg3 splice variants. (A)** Schematic illustration of the *Meg3* gene exon structure. Exons found in all Meg3 isoforms are shown in white, variable exons are shown in black. The location of the primers used to detect the different *Meg3* isoforms are marked by arrows. **(B)** GCTSCs were cultured with or without the addition of Aza and PBA before *Meg3* splice variants were amplified by PCR using primers located in different exons. PCR products were separated on a 1.6% agarose gel. Untreated MSCs served as controls. The structure of the main transcript is indicated. Additional splice variants appear as larger transcripts above the main product.

**Figure 6:**
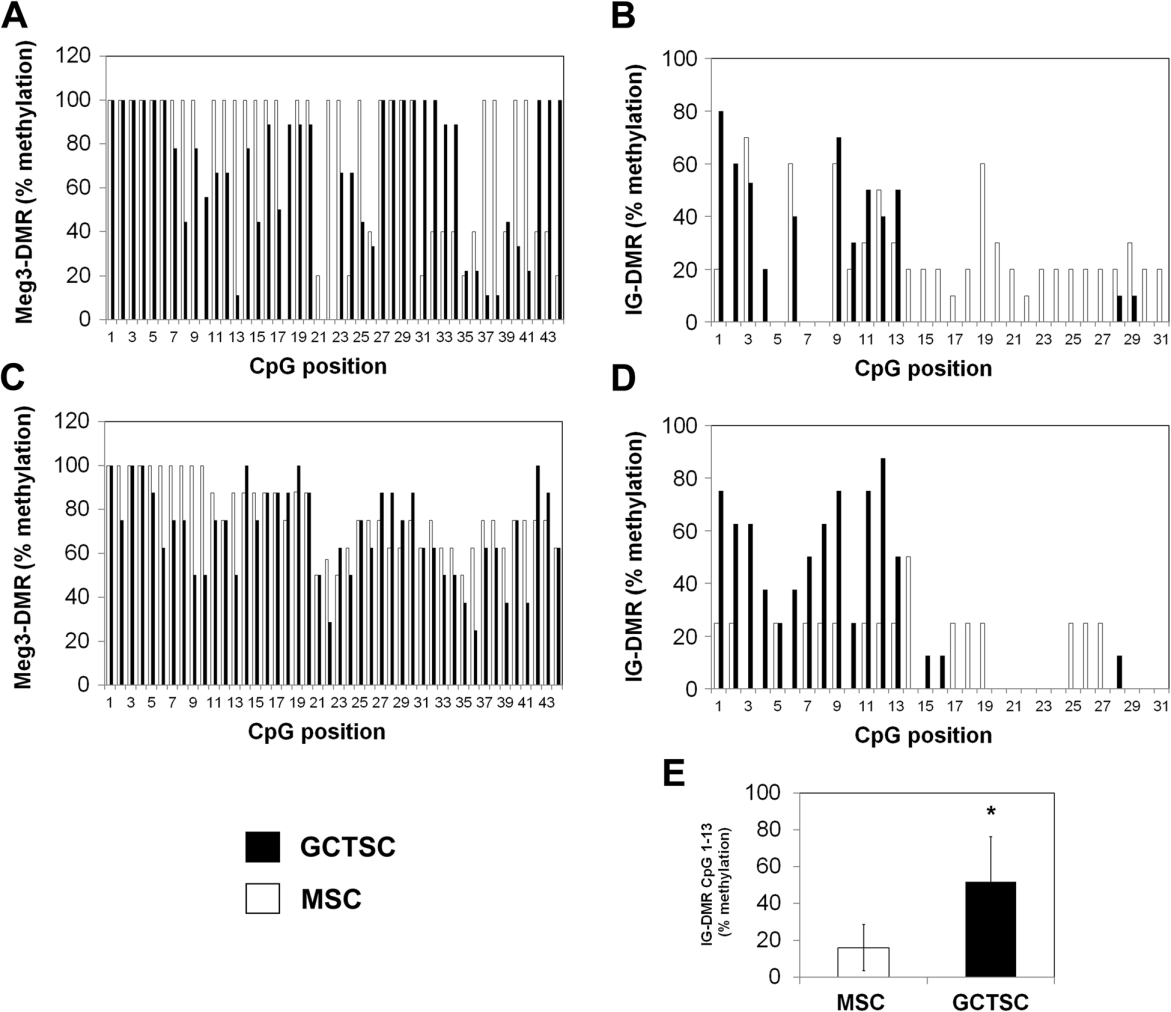
**Identification of a hypermethylated region within the IG-DMR of GCTSCs.** Cellular DNA was extracted from GCTSCs (n = 8) and MSCs (n = 8) and DNA fragments covering the Meg3-DMR (44 CpGs) and the IG-DMR (31 CpGs) were amplified by PCR, bisulfite treated, cloned into pCR4-TOPO vector and sequenced. **(A, B)** Calculated methylation frequencies of all analyzed CpGs within the Meg3-DMR and the IG-DMR of 10 individual clones derived from one GCTSC and one MSC cell line. **(C, D)** Calculated methylation frequencies within the Meg3-DMR and the IG-DMR of eight different GCTSC and MSC cell lines. **(E)** Methylation analysis restricted to the first 13 CpGs analyzed within the IG-DMR. Data are presented as mean ± SD. (*p < 0.05 determined by Mann–Whitney-*U* test).

In a previous study we investigated the microRNA expression profiles of GCTSCs and MSCs in order to identify possible candidates involved in the neoplastic transformation of MSCs during GCT pathogenesis. We could demonstrate that these two cell types differ in a microRNA signature consisting of only 26 differentially expressed microRNAs, mostly downregulated in GCTs. Interestingly, the coding region of five of these microRNAs is located within the Dlk1-Dio3 locus on chromosome 14 regulated by the differentially methylated regions IG-DMR and Meg3-DMR (Figure 1). RT-qPCR analysis showed a significant downregulation of these microRNAs in GCTSCs compared to MSCs (Figure 2). As the whole Dlk1-Dio3 region is known to be under the control of two differentially methylated regions, we assumed that GCTSCs and MSCs might also differ in gene expression patterns. In fact, we could detect a significant downregulation of *Dlk1* and the non-coding, maternally expressed genes *Meg3* and *Meg8* in GCTSCs. Although not significant, expression of *Rtl1* and *Dio3* was also reduced in GCTSCs (Figure 3A). A possible explanation for the observed differences in gene and microRNA expression might be chromosomal rearrangements, especially deletions within the Dlk1-Dio3 region. However, we could exclude this possibility by performing a DNA copy number assay based on real time PCR amplification and detection with an IG-DMR specific TaqMan probe. Two copies were detected in MSCs, GCTSCs and normal osteoblasts taken as controls (Figure 3B). To investigate the involvement of epigenetic mechanisms in the regulation of microRNA and gene expression we treated GCTSCs with the demethylating agent Aza (5-Aza-2′-deoxycytidine), the histone deacetylase inhibitor PBA (phenyl butyric acid) or a combination of both. Expression of the genes *Dlk1*, *Meg3*, *Meg8, Rtl1* and *Dio3* slightly increased after treatment with Aza but no significant differences could be detected. However, a considerable increase in gene expression could be induced by PBA. The combined treatment of GCTSCs with Aza and PBA further increased expression of all analyzed genes (Figure 4A). A significant but more selective influence of epigenetic modifiers could also be observed on microRNA expression levels. While expression of miR-136, miR-376a and miR-377 did not significantly change during treatment, expression of miR-376c and miR-127-3p was significantly increased by Aza treatment and was further elevated by the combined treatment with Aza and PBA. Interestingly, PBA alone had no effect on microRNA expression (Figure 4B). Notably, during RT-qPCR analysis of *Meg3* expression, we observed a different melting temperature of the amplification product in GCTSCs compared to MSCs, indicating the synthesis of a different DNA fragment. The *Meg3* gene contains 10 exons, while the original *Meg3* transcript identified in a human EST library is restricted to exons 1, 2, 3, 8, 9 and 10 [17]. Until now, at least 12 *Meg3* isoforms have been described that contain one or more of the additional exons 4–7 [18]. To analyze the expression of *Meg3* splice variants in GCTSCs we performed conventional PCR using forward primers located in exon 3, 4, 5 and 6 in combination with a reverse primer located in exon 8. In MSCs, that were taken as controls, primers located in exon 3 and 8 that should amplify all *Meg3* isoforms, produced a main fragment of 186 bp that corresponds to the isoform consisting of exons 1, 2, 3, 4, 8, 9 and 10. This isoform has already been shown to be the most abundant *Meg3* transcript in many other cell types [18]. In addition, larger fragments of additional isoforms could also be detected in untreated MSCs. In contrast, the main transcript is completely missing in untreated GCTSCs that only express very low amounts of some other splice variants, explaining the observed differences in the melting temperature during RT-qPCR analysis of untreated cells. Treatment of GCTSCs with epigenetic modifiers restored expression of all *Meg3* isoforms to comparable levels observed in untreated MSCs. Based on the location of the primer and the size of the PCR products, we could identify all known *Meg3* isoforms in GCTSCs treated with Aza and PBA (Figure 5A,B). Our data suggested that epigenetic mechanisms are involved in the observed downregulation of genes and microRNAs in GCTSCs. Thus, we aimed to investigate the degree of methylation within the IG-DMR and the Meg3-DMR in GCTSCs and MSCs. The methylation status of 31 CpG dinucleotides within a 909 bp DNA fragment covering the IG-DMR and 44 CpG dinucleotides within a 819 bp fragment covering the Meg3-DMR was investigated. Analysis was done using bisulfite sequencing of cloned DNA fragments. Methylation frequencies were calculated for each CpG as percent methylation in all analyzed samples. In a first step we analyzed 10 individual clones derived from one GCTSC and one MSC cell line. Within the analyzed Meg3-DMR region we could not detect any hypermethylation in the GCTSC cell line compared to MSC that could contribute to gene and microRNA silencing. Detected methylation frequencies were rather decreased in the GCTSC sample. However, we could detect elevated methylation frequencies within the range of the first 13 analyzed CpGs of the IG-DMR region in GCTSC compared to the MSC sample (Figure 6A,B). To validate these results we extended the analysis to eight different GCTSC and MSC cell lines and observed comparable methylation frequencies. A significant hypermethylation of CpGs 1–13 within the analyzed IG-DMR region was consistently detected in all GCTSC cell lines compared to MSCs (Figure 6C-E).

## Discussion

There is growing evidence that GCTSCs, the neoplastic cell population within GCTs, develop from MSCs. Particularly, the expression of mesenchymal stem cell markers and the observation of an osteoblastic, chondroblastic and adipogenic differentiation potential of GCTSCs support this hypothesis [6,7]. However, the molecular mechanisms involved in the neoplastic transformation of MSCs are largely unknown. In order to identify possible mediators of this progress we performed comparative gene and microRNA expression analysis of GCTSCs and MSCs obtained from the same patient in previous studies [8,9]. We identified a microRNA signature consisting of 26 microRNAs which clearly differentiates between GCTSCs and MSCs. Interestingly, 23 of these microRNAs are silenced or downregulated in GCTSCs and five of them are located within the imprinted Dlk1-Dio3 locus on chromosome 14q32. In addition to the paternally expressed genes *Dlk1*, *Rtl1* and *Dio3* and the maternally expressed genes *Meg3* and *Meg8* this region harbors one of the largest microRNA clusters in the human genome consisting of 54 microRNAs [19]. Aberrant expression of several microRNAs located within this region has been implicated in the pathogenesis of several tumors including esophageal squamous cell carcinoma [20], gastric cancer [21], gastrointestinal stromal tumor [13], colorectal cancer [22] and hepatocellular carcinoma [23]. At least eight microRNAs within this cluster have been identified as potential tumor suppressors, among them mir-376a and miR-377, silenced in GCT [9,11]. Besides alterations in microRNA expression also deregulations of gene expression within this chromosomal region have been observed in several types of tumors including neuroblastoma, pituitary adenomas, hepathocellular carcinomas and multipla myelomas [24-27]. For example, expression of the non-coding, maternally expressed gene *Meg3* has been shown to be lost in many kinds of primary human tumors and tumor cell lines. Re-expression of *Meg3* inhibits cell proliferation and induces apoptosis and accumulation of p53, thus, influencing the expression of p53 target genes. Therefore, *Meg3* is supposed to have tumor suppressor properties [28]. Likewise, tumor suppressor characteristics have been demonstrated for the paternally expressed gene *Dlk1*. In contrast to normal kidney tissue, loss of *Dlk1* expression has been shown in renal cell carcinoma and re-expression of *Dlk1* markedly increased anchorage-independent cell death and suppressed tumor growth in nude mice [29]. In agreement with these findings we could observe a significant downregulation of *Dlk1*, *MEG3* and *MEG8* expression in GCTSCs compared to MSCs. Together with our observation of microRNA silencing in GCTSCs, these data indicate that deregulations within the Dlk1-Dio3 locus are also involved in GCT pathogenesis and might play an important role in the malignant transformation of MSCs. With respect to the assumed development of GCTSCs from MSCs the observation of an involvement of the Dlk1-Dio3 locus in the regulation of cellular stemness is of particular importance. Gene and microRNA transcript levels have been shown to correlate with pluripotency status of induced pluripotent stem cells from mice [30,31]. Further, aberrant expression of specific microRNAs within this region has been attributed to a stem-like subtype of hepatocellular carcinoma associated with poor prognosis [23]. While frequently allelic loss (LOH) of chromosome 14q has been reported to be responsible for aberrant gene expression [32-34], epigenetic alterations have also been shown to influence gene and microRNA expression within this chromosomal region, mainly mediated by the differentially methylated regions IG-DMR and MEG3-DMR [12,26,35]. In GCTSCs we could not detect any copy number variations of the IG-DMR locus suggesting that predominantly epigenetic alterations are responsible for the observed downregulation of gene and microRNA expression. While methylation analyses of the Meg3-DMR could not reveal any hypermethylated regions that might be associated with gene and microRNA silencing in GCTSCs, we identified a region within the IG-DMR spanning 13 CpG dinucleotides that is frequently hypermethylated in GCTSCs compared to MSCs. Our observation of a restored gene expression after a combined treatment with the demethylating agent Aza and the histondeacetylase inhibitor PBA further confirmed the importance of epigenetic regulatory mechanisms within the Dlk1-Dio3 locus of GCTSCs. The fact, that PBA alone or in combination with Aza showed the most pronounced effects on gene expression suggests that, in addition to the identified alterations in DNA methylation, additional epigenetic mechanisms like histone modifications are involved in the regulation within the Dlk1-Dio3 region in GCTs. Further, we observed different effects of epigenetic modification on gene and microRNA expression. While all analyzed genes within the Dlk1-Dio3 locus responded to Aza and PBA treatment, the expression of only 2 out of 5 analyzed microRNAs was affected. In contrast to the analyzed genes, PBA alone had no effect on microRNA expression. These data suggest that in addition to the central role of the differentially methylated regions IG-DMR and Meg3-DMR additional regulatory elements must be present. Taken together, besides silencing of specific microRNAs we could demonstrate that further genes located within the Dlk1-Dio3 region are downregulated in GCTSCs compared to MSCs. We could identify a range of CpG dinucleotides within the IG-DMR that is frequently hypermethylated in GCTSCs and might thus contribute to the observed gene and microRNA downregulation. Treatment with epigenetic modifiers could restore gene and microRNA expression, but suggests further mechanisms involved in the regulation of this complex chromosomal region.

## Conclusion

Our data suggest that epigenetic silencing of genes and microRNAs within the Dlk1-Dio3 region is a common event in GCTSCs that is in part mediated by hypermethylation within the IG-DMR. However, further mechanisms seem to be involved in the regulation of this complex chromosomal region that have to be investigated. The identified genes, microRNAs and microRNA target genes might be involved in the neoplastic transformation of MSCs and thus represent valuable targets for the improvement of GCT diagnosis and therapy.

## Abbreviations

GCT: Giant cell tumor; GCTSC: Giant cell tumor stromal cell; MSC: Mesenchymal stem cell; FGFR1: Fibroblast growth factor receptor3; Dlk1: Delta-like homolog 1; Rtl1: Retrotransposon-like 1; Dio3: Iodothyronine deiodinase 3; Meg3: Maternally expressed gene 3; Meg8: Maternally expressed gene 8; RPL19: Ribosomal protein L19; CSFR1: Colony stimulating factor 1 receptor; DMR: Differentially methylated region; Aza: 5-Aza-2′-deoxycytidine; PBA: Phenyl butyric acid.

## Competing interests

The authors declare that they have no competing interests.

## Authors’ contributions

BL, PK and JF contributed in conception and design of the study. HS and JF performed the experiments and acquired the data. PK, HS and JF performed analysis and interpretation of data. BL supervised the study and provided financial support. JF drafted and wrote the manuscript. BL, PK and JF revised the manuscript. All authors read and approved the final manuscript.

## Pre-publication history

The pre-publication history for this paper can be accessed here:

http://www.biomedcentral.com/1471-2407/14/495/prepub
